# Mobile and Fixed Computer Use by Doctors and Nurses on Hospital Wards: Multi-method Study on the Relationships Between Clinician Role, Clinical Task, and Device Choice

**DOI:** 10.2196/jmir.1221

**Published:** 2009-08-04

**Authors:** Pia Andersen, Anne-Mette Lindgaard, Mirela Prgomet, Nerida Creswick, Johanna I Westbrook

**Affiliations:** ^2^Health Informatics Research and Evaluation UnitUniversity of SydneyAustralia; ^1^Medical Informatics GroupDepartment of Health Science and TechnologyAalborg UniversityDenmark

**Keywords:** Study, multi-method study, observational study, mobility, mobile computers, computers, computer hardware, medical order entry systems, computerized physician order entry system, computerized provider order entry (CPOE)

## Abstract

**Background:**

Selecting the right mix of stationary and mobile computing devices is a significant challenge for system planners and implementers. There is very limited research evidence upon which to base such decisions.

**Objective:**

We aimed to investigate the relationships between clinician role, clinical task, and selection of a computer hardware device in hospital wards.

**Methods:**

Twenty-seven nurses and eight doctors were observed for a total of 80 hours as they used a range of computing devices to access a computerized provider order entry system on two wards at a major Sydney teaching hospital. Observers used a checklist to record the clinical tasks completed, devices used, and location of the activities. Field notes were also documented during observations. Semi-structured interviews were conducted after observation sessions. Assessment of the physical attributes of three devices—stationary PCs, computers on wheels (COWs) and tablet PCs—was made. Two types of COWs were available on the wards: generic COWs (laptops mounted on trolleys) and ergonomic COWs (an integrated computer and cart device). Heuristic evaluation of the user interfaces was also carried out.

**Results:**

The majority (93.1%) of observed nursing tasks were conducted using generic COWs. Most nursing tasks were performed in patients’ rooms (57%) or in the corridors (36%), with a small percentage at a patient’s bedside (5%). Most nursing tasks related to the preparation and administration of drugs. Doctors on ward rounds conducted 57.3% of observed clinical tasks on generic COWs and 35.9% on tablet PCs. On rounds, 56% of doctors’ tasks were performed in the corridors, 29% in patients’ rooms, and 3% at the bedside. Doctors not on a ward round conducted 93.6% of tasks using stationary PCs, most often within the doctors’ office. Nurses and doctors were observed performing workarounds, such as transcribing medication orders from the computer to paper.

**Conclusions:**

The choice of device was related to clinical role, nature of the clinical task, degree of mobility required, including where task completion occurs, and device design. Nurses’ work, and clinical tasks performed by doctors during ward rounds, require highly mobile computer devices. Nurses and doctors on ward rounds showed a strong preference for generic COWs over all other devices. Tablet PCs were selected by doctors for only a small proportion of clinical tasks. Even when using mobile devices clinicians completed a very low proportion of observed tasks at the bedside. The design of the devices and ward space configurations place limitations on how and where devices are used and on the mobility of clinical work. In such circumstances, clinicians will initiate workarounds to compensate. In selecting hardware devices, consideration should be given to who will be using the devices, the nature of their work, and the physical layout of the ward.

## Introduction

The use of information and communication technologies (ICT) in the health care sector has become widespread in several countries [1], and governments around the world continue to invest in the implementation of ICT systems [2]. These clinical systems comprise a variety of functions, including medication management, order entry, results viewing, clinical documentation, and decision support capability [3]. Introduction of ICT in a hospital affects the operation of the organization, health care delivery, and patient outcomes while offering potential benefits in improved patient safety, reduced hospital costs, and increased efficiency and effectiveness of medical care [4-6].

Not all ICT systems are successfully implemented. A key factor for success is the extent to which systems integrate with clinical workflow [7,8]. Clinical work is characterized by a complex mixture of routine and unexpected events and involves close collaboration among practitioners [9]. Furthermore, clinical work is highly mobile. Health professionals move frequently among wards, clinics, offices, and other locations and require information at each of these locations [10,11]. Thus, new ICT systems must, among other factors, complement the mobile, collaborative nature of medical work in order to succeed [9].

A core component of system implementation is the selection of hardware. Early clinical system implementations relied upon replacing paper-based records with information accessible via stationary personal computers (PCs). Stationary PCs allow easy storage, searching, retrieval, and sharing of information [12]; however, they constrain work to a fixed location [13]. The design of new and more mobile hardware devices has increased the range of options available to health care organizations, and mobile devices have been advocated [14] as a means of providing practitioners with access to patient and clinical information at the point of care [15]. Subsequently, the integration and use of both stationary and mobile computing technologies within health care have been promoted as the approach most likely to achieve the greatest results for clinicians and their patients [13,16].

Systematic reviews of the use of handheld devices within medicine have reported benefits in allowing easy access to information, decision support, and improved communication, but such reviews have also identified barriers to their implementation and use [17-21]. Very few studies have made comparisons between stationary and mobile devices, investigated how clinical staff select a device when multiple devices are available, or determined the locations in which clinicians choose to use mobile devices. Thus, this limited research base means that determining the right mix of stationary and mobile devices is a significant challenge for system planners and implementers. Little is known about the degree to which different hardware devices are capable of adequately supporting the complex and often collaborative nature of work in a hospital environment. In order to obtain the benefits ICT systems offer, it is necessary to gain insights into the way users interact with different hardware devices and the impact these systems have on work practices. The extent or ways in which different hardware devices may be more effective for different professionals or clinical tasks has rarely been investigated.

We conducted a study to investigate the relationships between clinician role, clinical task, and selection of hardware device. Our aim was to answer two central questions: (1) which device is used by whom, where, for which clinical task, and in collaboration with whom? and (2) what impact does the design of the device have on its use on hospital wards?

## Methods

### Study Design

We utilized a multi-method approach which included: (1) direct observations, (2) interviews, (3) an assessment of the physical attributes of available hardware devices, and (4) heuristic evaluation of the user interfaces. Advocates of a multi-method design support its use as a means of gaining clearer understanding and insights into the impact of technology on health care services [22].

### Setting

The study was conducted in two geriatric wards of a Sydney metropolitan teaching hospital. Each ward had 26 beds and was at maximum occupancy throughout the majority of the study period. The Cerner Millennium PowerChart system (Cerner, Kansas City, MO), which comprises computerized test ordering, results viewing, and electronic medication management, was available from stationary PCs, COWs, and tablet PCs across both study wards. While computerized test ordering and results viewing have been used for several years in the two wards, the electronic medication management system had been implemented and used since November 2007 in ward A and July 2008 in ward B.

### Computer Hardware Devices and Ward Layouts

**Figure 1:**
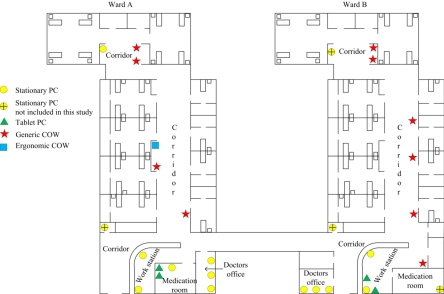
Overview of the placement of devices in ward A and ward B

**Figure 2:**
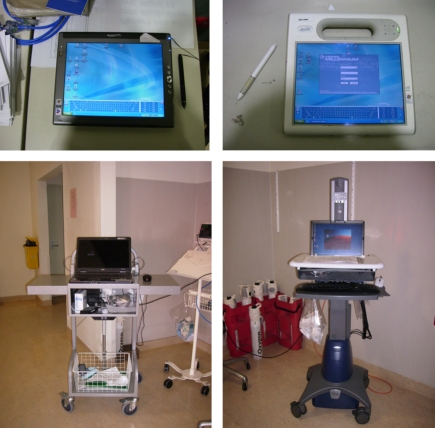
Devices available in the two wards (A: tablet PC ward A, B: tablet PC ward B, C: generic COW, D: ergonomic COW)

The two wards were located next to one another, and their layout and characteristics were much alike (Figure 1). Each ward had seven stationary PCs. Three were located in the doctors’ offices, one in the corridor, one in the medication room, and two in the central workstation (nurses’ and clerks’ station). The two wards also had stationary PCs in the Nursing Unit Manager’s office; however, these computers were not included in the study as they were not used for clinical tasks. Ward A and ward B had five and six COWs, respectively. In ward A, four of the COWs were generic COWs (laptops mounted on trolleys—three Acer Travelmate 5620 and one Acer Travelmate 7720), and one was an ergonomic COW (an integrated computer and cart device specifically designed to make it easy to move—Dell Latitude D620 and StyleView® Notebook Cart) (Figure 2). In ward B, the six COWs were all generic COWs (Acer Travelmate 7720). The COWs were generally distributed throughout the corridor area. Each of the wards also had two tablet PCs (two Motion Computing C5 and two Motion Computing LE1700). In ward A, the tablet PCs were placed in the medication room, while in ward B they were kept at the central workstation. All devices ran Windows XP as their operating system.

### Sample

The observational study was conducted between October 13 and 31, 2008 for a total of 80 hours and 1 minute spread across 144 observation sessions. The sample consisted of 27 nurses (13 from ward A and 14 from ward B) and 8 doctors who worked across both wards. This represents approximately 50% of the nursing and medical staff allocated to these wards. Nurses had an average work experience of 2.7 years in the respective wards, and the average experience of doctors was 2.9 years in the study wards. Participants were provided with an information letter outlining the study, and each consenting participant was assigned a unique identifying number. When the researchers arrived on the ward, participants using the PowerChart system were identified and one was selected for observation. Each participant was observed for a maximum of two consecutive hours and a maximum of six hours in total for the duration of the study. A convenience sample of ten nurses and two doctors who participated in the observational study also participated in brief interviews.

### Data Collection Procedures

#### Observational Study

The observational study was conducted over a three-week period in October 2008. The study participants were observed while undertaking clinical tasks during their day-to-day routine that required interaction with a stationary PC, one of two styles of computers on wheels (COWs), or a tablet PC.

We developed an observational data collection form with a list of clinical tasks based upon previous work (Table 1) [23,24]. This form was used to record: the participant-ID; the ward; the date; the start and end time of each observation session; clinical tasks performed; hardware device used (stationary PC, generic COW, ergonomic COW or tablet PC); the location of task completion (doctors’ office, corridor, bedside, medication room, workstation, or patient room); whether collaboration occurred during the task (with a doctor, nurse or other health care personnel); and whether the observed participant was on a ward round. The location was defined as at the “bedside” if the observed participant used one of the devices directly next to the bedside of the patient being treated. Where the device was placed next to one patient’s bedside while treatment was provided to another patient within the room, the location was recorded as “patient room”. Collaboration between the observed participant and another health professional was recorded if they looked at the computer screen at the same time and conducted a clinical task together (eg, clinicians discussing a particular prescription in the medication chart or a nurse observed asking another nurse to document witnessing a drug to be administered). During the observation sessions, additional factors potentially impacting on device selection were also noted, for example, when a computer crashed. The average length of the observation sessions was 33 minutes. All observational sessions were conducted by AL and PA.

**Table 1:** Clinical task classification

Clinical task	Definition
Review chart	Reviewing the medical chart, allergy, height, weight, health insurance, or other patient information.
Prepare drug	Reading medication order to select drug.
Administer drug	Recording that a drug has been administrated.
Unchart drug	Removing documentation of drug administration (eg, because it was recorded prior to actual administration and the drug did not end up being given to the patient).
Witnessing drug	Confirming that preparation of a restricted drug, or another special drug, has been checked.
Ordering drug/test	Writing up a new medication order or ordering a test.
Changing/canceling drug	Changing drug order details or canceling a drug order.
Viewing results	Looking at a patient’s test results.
Generating discharge	Writing a discharge summary.
Reorder drug	Extending the length of a drug order.
Drug information	Looking at drug information.
Documenting allergies	Documentation of a patient’s allergies.
Documenting others	Documentation of other information relevant to medication orders (eg, height, weight, blood glucose).
Copying medication order	Copying medication orders from paper to PowerChart.

Researchers were trained in the PowerChart system to allow them to become acquainted with the system and to be able to distinguish accurately among the different clinical tasks performed using the system. A pilot study, consisting of approximately 17 hours of observation, was undertaken between October 7 and 13, 2008 to assess the design of the data collection form. During the first session of the pilot study, it was discovered that the doctors and nurses changed clinical tasks rapidly, which was not supported by the initial design of the form. The pilot study also revealed that the list of defined clinical tasks did not cover all tasks doctors and nurses conducted in the PowerChart system, and some of the defined tasks were difficult for the researchers to distinguish. This led to the addition of new tasks (eg, “unchart drug”) and combination of other tasks (eg, entering a new drug and ordering a test were combined in the task “ordering drug/test”). After modifying and testing the new data collection form, inter-observer reliability testing between the researchers was conducted by the two observers concurrently, but independently, observing nurses and doctors for a total of four hours and comparing their results. Formal data collection began once an overall agreement of over 85% was achieved between the data collectors. Throughout the study, data were collected on weekdays between the hours of 7 am and 4 pm. Each researcher spent half of the study period independently observing in one ward and then rotated to observe in the alternate ward. After each observation session, the manually collected data were transcribed onto a computer. Field notes were documented by the researchers to provide contextual information about, for example, physical space limitations which impacted upon the way a device was used.

#### Interviews

Interviews were conducted during the last week of data collection. Based on the prior observations, the nurses and doctors were asked about their individual device preference in a given context (eg, when giving medication or going on ward rounds) and their reasons for choosing a particular device. Participants were asked the following questions: When conducting a task in relation to medication, results viewing, or ordering tests, which of the hardware devices do you generally use and why?; Is there a certain device you prefer and why?; Do you ever use one of the other devices for certain tasks? If yes, what made you choose to use the other device? If no, has somebody shown you how to use the device? If yes, why have you decided not to use that device? The data collected in the interviews were recorded on paper and later transcribed to digital format. The interviews were conducted by the same researchers who undertook the observations.

#### Physical Attributes Assessment

Examination of the physical attributes of each hardware device (such as the size of the screen and boot-up time) was performed to further identify factors that may impact on device selection. These were conducted by two researchers (AL and PA). A form was developed to evaluate the physical attributes of each device. Weighing the generic and ergonomic COWs was not possible; therefore, only the tablet PCs were weighed. The system boot-up times were measured, as were the start and restart times. The start time was calculated from the time the start button on the device was pressed until the programs had finished loading (indicated by the hourglass disappearing). The time it took to restart the device was measured from pressing “ok” on the Windows shut-down screen until the device had finished reloading all programs. Additionally, the time it took to start PowerChart was measured. First, the time from clicking on the PowerChart icon on the desktop until the login screen appeared was measured. Second, after typing in a username and password, the time from pressing “ok” on the log in screen until PowerChart had completely loaded was measured. These times were measured both immediately after the computer had been started or restarted (one time) and after PowerChart had been previously opened (measured three times). Each device within the wards was evaluated individually.

#### Heuristic Evaluation

The heuristic evaluation was undertaken to identify design factors which may influence device selection and use. The evaluation was completed independently by two researchers (AL and PA) for each defined clinical task and conducted on the basis of a set of 10 recognized usability principles [25]. Following independent assessment by each investigator, results were compared with regard to how clinical tasks were completed, taking into account the 10 usability principles to gain consensus. As the same clinical system was accessible across the various computing devices and the appearance of the system was identical, the particular focus was on identifying how the screen size of each device may have affected the aesthetics and usability of the device, subsequently influencing device selection. The evaluation was conducted on a stationary PC with a 17″ screen followed by a tablet PC with a 10.5″ screen, with an emphasis on user-interface design issues that might be present in smaller devices.

### Data Analysis

#### Observational Study and Interviews

Observational data were highly structured as described above, and data analysis was undertaken in SPSS version 16.0 (SPSS Inc, Chicago, IL). Descriptive statistics were calculated, including the proportions of clinical tasks undertaken using the different devices, device use by clinical group, and the physical locations where devices were used. Interviews and field notes were analyzed separately by the researchers and the main themes identified. This involved, for example, all responses to specific interview questions being independently reviewed by two researchers, and the most frequently recurring issues raised by respondents were identified. This allowed identification of the factors which doctors and nurses most frequently raised as reasons for why they selected or did not select a specific device. Given the structured nature of the questions, there was little disagreement between reviewers. When disagreement arose, it was resolved through consensus and discussion with the other co-authors. Field notes were analyzed in terms of identification of the range of issues raised with greater emphasis given to those factors which occurred on multiple occasions. For re-occurring issues, these were counted to provide an indication of their frequency.

#### Physical Attributes Assessment and Heuristic Evaluation

Physical and heuristic assessment data were tabulated to allow comparisons between devices on a range of attributes. The physical attributes data were used to calculate the mean and standard deviation boot-up times for each device.

The study was approved by the University of Sydney Human Research Ethics and the hospital Human Research Ethics Committees.

## Results

### Observational Study Results

A total of 144 observation sessions was conducted over a period of 80 hours and one minute during which 2158 clinical tasks were observed. Nurses were observed in 103 (71.5%) of the observation sessions, and their tasks accounted for 81.1% of the tasks recorded. Doctors were observed in 41 (28.5%) observation sessions, and their tasks made up 18.9% of the tasks observed. Of the observed tasks performed by doctors, 57.5% were collected during ward rounds.

The number of clinical tasks performed per individual ranged from 3 (0.1%) to 227 (10.5%) for nurses and 7 (0.3%) to 112 (5.2 %) for doctors. Observations were fairly evenly distributed across the two wards, with 71 sessions conducted in ward A, in which 42.6% of the tasks were collected, and 68 sessions in ward B, accounting for 57.4% of tasks. Five observation sessions occurred in both wards, where doctors walked from one ward to the other and used a hardware device in both wards.

#### Observed Use of Hardware Device by Clinical Task and Location

The majority of observed tasks (82.3%) were undertaken using the generic COW, with 9.6% conducted on a stationary PC, 5.1% on a tablet PC, and 3.0% on the ergonomic COW. Most clinical tasks (49.1%) were completed in a patient’s room, followed by the corridor (35.2%), the doctors’ office (8.1%), a patient’s bedside (4.3%), the central workstation (3.0%), and the medication room (0.2%). The most frequently observed tasks were administering drugs (31.4%), preparing drugs (27.9%), and reviewing a patient’s chart (15.4%). The frequency of the other observed tasks ranged from 0.1% to 7.5%.

#### Observation of Nurses’ Use of Hardware Devices on Wards

Nurses undertook 93.1% of observed tasks using the generic COWs. All except one of the 27 observed nurses used a generic COW during the study period. Ten nurses also used a stationary PC, four nurses used an ergonomic COW, and one nurse used a tablet PC during the study period.

The generic COWs, ergonomic COW, and the tablet PCs were most frequently used by nurses in the patients’ rooms (56.7%) and in the corridors (35.8%), mostly to administer and prepare drugs. If a generic COW and the ergonomic COW were available and placed next to each other, it was often observed that nurses chose the generic COW. When nurses used the stationary PCs, they primarily used the ones at the workstations, followed by those located in the corridors, to witness and prepare drugs (Table 2). Less than 2% of nurses’ tasks were undertaken using stationary PCs. None of the nurses were observed using a stationary PC in the doctors’ office or in the medication room. It was observed on one occasion that a generic COW was wheeled into the medication room, when a stationary PC within the room was out of order.

**Table 2:** Clinical tasks conducted by nurses by device and location of use (N = 1751 tasks)

Location	Clinical task	Tablet PC	Generic COW	Ergonomic COW	Stationary PC	Total (% of total tasks)
**Bedside**						
	Review chart	1	5	-	-	6
	Prepare drug	1	28	1	-	30
	Administer drug	3	30	2	-	35
	Witnessing drug	1	6	1	-	8
	Ordering drug/test	-	1	-	-	1
	Drug information	-	1	-	-	1
	Viewing results	-	1	-	-	1
	Documenting others	-	3	1	-	4
	Total	6	75	5	-	86 (4.9%)
**Patient’s room**						
	Review chart	-	65	4	-	69
	Prepare drug	4	348	10	-	362
	Administer drug	6	396	11	-	413
	Unchart drug	-	7	1	-	8
	Witnessing drug	3	62	3	-	68
	Ordering drug/test	-	14	2	-	16
	Changing/canceling drug	-	1	-	-	1
	Reorder drug	-	1	-	-	1
	Drug information	-	12	1	-	13
	Viewing results	-	2	-	-	2
	Documenting others	1	38	-	-	39
	Total	14	946	32	-	992 (56.7%)
**Medication room**						
	Prepare drug	-	2	-	-	2
	Administer drug	-	2	-	-	2
	Ordering drug/test	-	1	-	-	1
	Total	-	5	-	-	5 (0.3%)
**Workstation**						
	Review chart	-	1	-	1	2
	Prepare drug	-	1	-	5	6
	Administer drug	-	8	-	6	14
	Witnessing drug	-	8	-	10	18
	Total	-	18	-	22	40 (2.3%)
**Corridor**						
	Review chart	2	96	10	2	110
	Prepare drug	2	187	9	3	201
	Administer drug	1	203	6	1	211
	Unchart drug	-	6	-	-	6
	Witnessing drug	2	63	2	-	67
	Ordering drug/test	-	9	-	-	9
	Reorder drug	-	1	-	1	2
	Drug information	-	7	-	-	7
	Viewing results	-	1	-	-	1
	Documenting others	-	11	1	-	12
	Other task	-	1	-	-	1
	Total	7	585	28	7	627 (35.8%)
**Multiple locations**						
	Prepare drug	-	1	-	-	1
	Total	-	1	-	-	1 (0.06%)
**Total**		27	1630	65	29	1751 (100%)

#### Observation of Doctors’ Use of Hardware Devices on Ward Rounds and Outside Ward Rounds

Eight doctors were observed at least once on a ward round during which they primarily used a generic COW (Table 3). Four doctors were observed using a stationary PC, and two doctors used a tablet PC, while none of them used an ergonomic COW on ward rounds during the observation period.

Of the observed tasks conducted on ward rounds, the majority (57.3%) were completed using a generic COW, while 35.9% were completed using a tablet PC. These two devices were most frequently used in the corridor, followed by the patients’ rooms, to review charts and view results. Use of a tablet PC at the workstation was observed more often than the use of a stationary PC (Table 3). Of the 234 clinical tasks completed during ward rounds, 57.3% were undertaken in the corridors, 29.1% in the patients’ rooms, and 3% at a patient’s bedside.

Seven doctors were observed at least once while not on a ward round. On each of these occasions they were observed using a stationary PC, with one doctor also using a generic COW. Doctors were not observed using tablet PCs or the ergonomic COW when not on ward rounds.

These seven doctors (not on ward rounds) conducted the vast majority (93.6%) of tasks using stationary PCs to view results and review charts, and most often they did this within the doctors’ office (Table 3). Generating discharge summaries was primarily conducted when doctors were not on ward rounds and only using stationary PCs.

**Table 3:** Clinical tasks conducted by doctors on ward rounds and not on ward rounds by device and location of use (n = 407 tasks)

			Device			
Location		Clinical task	Tablet PC	Generic COW	Stationary PC	Total (% of total tasks)
**Ward round**						
	Bedside					
		Review chart	2	-	-	2
		Changing/canceling drug	1	-	-	1
		Viewing results	4	-	-	4
		Total	7	-	-	7 (1.7%)
	Patient’s room					
		Review chart	10	15	-	25
		Ordering drug/test	9	5	-	14
		Changing/canceling drug	2	4	-	6
		Viewing results	10	13	-	23
		Total	31	37	-	68 (16.7%)
	Doctors’ office					
		Review chart	-	-	3	3
		Ordering drug/test	-	-	4	4
		Changing/canceling drug	-	-	2	2
		Generating discharge	-	-	1	1
		Viewing results	-	-	3	3
		Total	-	-	13	13 (3.2%)
	Workstation					
		Review chart	4	-	1	5
		Ordering drug/test	1	-	1	2
		Viewing results	6	-	-	6
		Other task	-	-	1	1
		Total	11	-	3	14 (3.4%)
	Corridor					
		Review chart	16	38	-	54
		Administer drug	-	1	-	1
		Ordering drug/test	4	12	-	16
		Changing/canceling drug	6	15	-	21
		Drug information	-	1	-	1
		Viewing results	9	30	-	39
		Total	35	97	-	132 (32.4%)
	Total		84	134	16	234 (57.5%)
**Not on Ward Round**						
	Doctors’ office					
		Review chart	-	-	51	51
		Administer drug	-	-	2	2
		Ordering drug/test	-	-	22	22
		Changing/canceling drug	-	-	5	5
		Generating discharge	-	-	19	19
		Reorder drug	-	-	1	1
		Viewing results	-	-	61	61
		Other task	-	-	1	1
		Total	-	-	162	162 (39.8%)
	Workstation					
		Review chart	-	5	-	5
		Ordering drug/test	-	2	-	2
		Viewing results	-	4	-	4
		Total	-	11	-	11 (2.7%)
	Total		-	11	162	173 (42.5%)

#### Collaborative Activities by Nurses and Doctors Using Hardware Devices

Nurses collaborated with other health professionals on 197 clinical tasks (11.3%) undertaken while using a computer device. Nurses mostly collaborated with other nurses (58.4%) and student nurses (40.0%) using generic COWs (91.9%). Collaboration centered around the witnessing (33.5%, n = 66), preparation (27.4%, n = 54) and administration (24.9%, n = 49) of drugs. Nurses were observed collaborating with a doctor using generic COWs to review charts (2 tasks) and prepare drugs (1 task).

Doctors on ward rounds conducted 88 clinical tasks (37.6%) in collaboration with another health professional. On ward rounds, doctors collaborated most often with other doctors (68.2%) and medical students (26.1%), and this occurred most frequently using a generic COW (75.0%). Doctors most often collaborated on ward rounds to review charts (42.0%, n = 37) and view results (39.8%, n = 35). Doctors not on ward rounds conducted 12 clinical tasks (6.9%) in collaboration with another health professional and mostly collaborated with other doctors (58.3%) on the stationary PCs (100.0%) and primarily when viewing results (50%, n = 6). Doctors were not observed collaborating with nurses at any time when using one of the computing devices.

#### Contextual Factors Identified From the Field Notes

Two main themes from the field notes were identified: device mobility and availability. In relation to mobility, nine nurses placed a drawer containing a patient’s medications on top of the trolley of the generic COW. This was observed in 14 nurse observation sessions (13.6%). In this hospital, most patients’ medications are locked in their individual bedside tables. Thus, we observed nurses placing these medication drawers (one patient at a time) onto the generic COWs in both the patient’s room and also when the trolley was situated in the corridors. The nurses went back and forth between the device and each patient’s bedside to administer the medication.

During six observation sessions, five nurses and one doctor were observed writing details about a patient’s drug order on a napkin or a piece of paper. Most frequently this action was observed in patients’ rooms when using a generic COW. The nurses used the paper note to take with them to the medication room in order to prepare a medication which was not available in a patient’s bedside drawer. It was also observed that the generic COW was placed between a patient’s bed and a wall on several occasions which left clinicians with very little space for movement.

The field notes identified that availability of the hardware devices was also an issue. For example, in 15 sessions (10.4%), problems with the generic COWs occurred primarily because the device ran out of power due to the fact it had not been recharged or because it did not work when first attempted by the observed participant. Similar problems were observed with the ergonomic COW during four sessions.

### Results of Interviews With Nurses and Doctors

Ten of the 27 observed nurses and two of the eight observed doctors were interviewed regarding their choice of device for completing tasks in relation to medications management, results viewing, or test ordering. All interviewed nurses preferred to use a generic COW over a tablet PC when giving medication, and three of the nurses reported that they had never used a tablet PC. The two doctors had both used the tablet PC and generic COW on ward rounds, but one preferred the tablet PC while the other preferred the generic COW.

From the responses to the interview questions, which focused on why doctors and nurses selected or did not select specific devices, six main themes were identified. These were: availability, speed, mobility, device design, knowledge about the device, and problems. Eight participants (seven nurses and one doctor) stated that one of the main reasons for using the generic COW as opposed to the tablet PC or stationary PC was the design of the trolley, which had space for the storage of medication, paper charts and/or other equipment. Despite comments that the need to recharge the batteries of generic COWs was a limitation and restricted mobility, a larger number of users stated that the advantage of the generic COWs over the other devices was better mobility because they can be used everywhere and because COWs allow items required for task completion to be stored on them. In relation to the use of the tablet PCs, clinicians reported difficulties with the stylus, including that it made task completion slow. Other limitations included small screen size and that it was awkward to carry the tablet PCs while walking.

Nine participants (seven nurses and two doctors) stated that they had used the tablet PCs in their daily routine. Of the participants that had used a tablet PC, two had received training while four reported having never received training; in addition, three didn’t comment on whether they had received training. Five nurses only used the tablet PCs if no generic COWs were available or the generic COW had crashed. Three nurses stated they had not used the tablet PCs, and also mentioned that they had not received any training in the use of the tablet PC. Four nurses reported that they also used stationary PCs when preparing or witnessing drugs in the medication room as it is at the point of need.

### Results of Assessment of the Physical Attributes of Hardware Devices

The evaluation of the physical attributes revealed that none of the devices automatically logged off the PowerChart system or closed down after a period of inactivity. Additionally, it was possible to log on to the PowerChart system from more than one device at the same time. All devices were connected to the Internet through the hospital’s wireless network.

There were different types of stationary PCs in the two wards. Six of these had a 15″ screen, four had a 17″ screen, and two had a 19″ screen (Table 4). In ward B, the stationary PC in the medication room and the one at the end of the corridor were out of order throughout the data collection period, and thus the attributes of these devices were not included in the study.

**Table 4:** Physical attributes of the different devices

		Stationary PCs	Generic COW	Ergonomic COW	Tablet PC
	Number of devices	12	10	1	4
	Screen size diagonal (inch)	15'', 17'', 19''	17''	14''	10.5'', 12''
	Weight (kg)				1.6, 1.9
**Dimensions**			Trolley	Trolley	Device
	Height min/max (cm)	-	73/105 - 77/100	70/110	25 - 26
	Width min/max (cm)	-	92/121 - 96/126	53	29.5 - 25.5
	Depth (cm)	-	37 - 45	67	2.5 - 2.5
**Boot-up times (min)**						
	Mean restart time (SD)	2.54 (1.33)	2.39 (0.46)	2.13 (-)	3.51 (0.41)
	Mean start time (SD)	2.16 (1.35)	2.15 (0.50)	1.30 (-)	3.11 (1.12)
**Time from desktop to log on screen (sec)**								
	Mean 1^st^ time (SD)	20.81 (6.62)	17.23 (2.50)	26.16 (-)	20.68 (7.42)
	Mean subsequent times (SD)	16.12 (2.66)	16.05 (1.12)	18.21 (2.94)	15.06 (1.45)
**Time from log on screen to system start up (sec)**								
	Mean 1^st^ time (SD)	3.56 (0.57)	3.99 (0.57)	3.37 (-)	3.84 (0.6)
	Mean subsequent times (SD)	3.5 (0.45)	3.67 (0.48)	5.30 (2.64)	3.6 (0.51)

All generic COWs had a 17″ screen laptop placed on top of a trolley that was adjustable in height (from floor to the keyboard) and width (Figure 2, C: generic COW). There were two types of trolleys and each ward had one type. The ergonomic COW had a 14″ screen integrated onto a cart, which was only adjustable in height (Figure 2, D: ergonomic COW).

There were two types of tablet PCs available in the wards (Figure 2, A: tablet PC ward A and Figure 2, B: tablet PC ward B). The tablet PCs in ward B (Figure 2, B: tablet PC ward B) were designed with a handle to facilitate transportation, which was done by hand. The lowermost part of the screen on both types of tablet PCs contained an onscreen keyboard where the user, by means of a stylus, interacted with the PowerChart system.

### Results of Heuristic Evaluation

The main user interface was composed of a topmost header containing the name of the patient and the patient’s medical record number. The remaining part of the user interface comprised tabs, from where it was possible to access the functionality defined for each clinical task (eg, the medication administration record [MAR] tab contained detailed information about the patient’s medication). In some of the tasks, an additional window appeared. For example, drug information could be reached by right clicking on an icon near a specific drug in the MAR tab and choosing “reference manual”. A new window then appeared which contained information about the drug.

The main difference between using the PowerChart system on a 17″ screen and a 10.5″ screen on the tablet PC was that the system was adjusted to a smaller screen size, and as a result, the font size and the amount of patient data shown on the screen decreased. This resulted in the user having to scroll more with the stylus on a tablet PC to obtain an overview of the information presented on the screen. Additional usability problems were discovered in the task “drug information”. In the pop-up window containing drug information an “ok” button was placed in the bottom right corner, which was the only button available for closing the window and returning to the main user interface. When the drug information window was opened on a tablet PC, the lower part of the window was hidden behind the onscreen keyboard and the window needed to be dragged to a higher position before it was possible to close it. Additionally, it was only possible to move or reduce the size of the window by dragging in the frame of the window with the stylus, which was inconvenient. This usability problem was not present on the evaluated stationary PC.

## Discussion

This is one of the first studies to investigate computer hardware selection and to quantify the frequency of use by clinician role, clinical task, and location of use. We found that the choice of device was related to clinician role, clinical task, degree of mobility required, and device design. Nurses’ work, and tasks performed by doctors during ward rounds, requires highly mobile computer devices. Nurses and doctors on ward rounds showed a strong preference for generic COWs over all other devices. While the greater availability of the generic COWs would account for some of their high utilization, preference for the generic COWs was both observed by the researchers and reported by the clinicians. The ergonomic COW was used occasionally but often only when the generic COWs were unavailable. Rarely did nurses elect to use a tablet PC or a stationary PC.

### Nurses’ Use of Computer Devices

The nurses indicated that they preferred the generic COW due to its availability, mobility, and design. When not in use the generic COWs were placed along the corridor and were easily accessible to the nurses. The nurses also complimented the mobility of the device, in that it was easy to move around and could be used almost everywhere. The design of the generic COW, which allowed users to store medications and charts, was highly appreciated by the majority of nurses interviewed.

For nurses a high proportion of clinical tasks were completed in the patients’ rooms and in the corridors. Space in the patient rooms was a problem when using the generic COW and appeared to be a significant factor preventing nurses from using the COWs at the patients’ bedsides. During the observations, we noted that when the generic COW was placed near a patient’s bed, it was often difficult for nurses to access the patient’s medication drawer. This resulted in the practice of taking the patient’s drawer out of the cabinet and placing it on top of the COW. This was a workaround in response to the space limitations imposed by the use of the COWs.

Although the concept of the ergonomic and generic COWs was identical (ie, a laptop placed on a trolley), our study showed large differences in user satisfaction with the two versions of the COW. The main disadvantage of the ergonomic COW was that the available table space was in front of the screen. When nurses placed items on the table space, these items obscured the screen. Additionally, the ergonomic COW had a 14″ screen, whereas the generic COWs had a 17″ screen. The ergonomic COW was also reported as being “hard to push around”. These findings clearly indicate that the design of mobile devices impacts their use, which has also been found in different settings [26]. Krogh et al [27] compared a generic COW with a tablet PC, in relation to supporting pharmacists’ clinical documentation. Based on subjective evaluations, the authors showed that the pharmacists preferred the tablet PC over the generic COW. The tablet PC was favored due to the design because pharmacists found the device easy to manoeuvre during rounds and the input system (handwriting-to-text functionality) user-friendly and simple.

Only one nurse in our study was observed using the tablet PC while giving medication. This nurse placed the tablet PC on an unused bedside table and rolled this around in the patient room almost as a substitute for a COW. Nurses reported a lack of obvious places to set down the tablet PC while providing direct patient care as a limitation. This problem was also identified by Bogossian et al [28], who found that the portability of tablet PCs was not viewed favorably. The authors reported that the tablet PC was placed at a central point and nurses kept coming back to it since it was inconvenient to carry around. In these situations, the generic COW, with the available trolley space, supports work routines in a better way and is one of the central reasons why the majority of nurses chose to use the COWs in preference to the tablet PCs.

### Doctors’ Use of Computer Devices

Doctors also preferred the generic COWs for highly mobile tasks such as conducting ward rounds. The generic COWs were preferred particularly on account of the trolley, with table and storage space, and the larger screen size which easily allowed more than one person to view the screen. The doctors also utilized the tablet PCs on many occasions; however, only the tablet PCs from ward B were used. This suggests that the design of the tablet PCs on ward B was preferable to the design of those on ward A. Tablet PCs on ward B weighed less and had a handle which made it easy to transport. Interestingly, when doctors used the COWs and tablet PCs the majority of this use occurred in the corridors, with one-third occurring in the patients’ rooms, and only a small proportion at the bedside. The benefit of mobile devices at the patient’s bedside was articulated by one doctor who commented that *“you can order and change medications and tests while you talk to and about the patient”*. In a study by Reuss et al [11], where clinical work routine was investigated, physicians reported that, if they did not enter information into the system right away, they felt like they had to process the job twice. Given that these devices are designed to enable use and provision of information directly at the point of care, it is surprising that they were not used at these locations in more instances. Unlike doctors on ward rounds, doctors not on ward rounds were generally not required to examine patients and found it more convenient to use the system on a stationary PC.

### Physical Attributes of Computing Devices Influencing Their Selection and Use

The stylus and the small screen size of the tablet PCs caused usability problems where scrolling was necessary due to the screen size but cumbersome to perform with the stylus. These factors also contributed to nurses selecting devices other than tablet PCs. Similar problems have been reported by doctors using laptops [29], and these factors have also been identified in other clinical settings [30]. Lack and type of training could also explain the low rates of use of the tablet PCs by nurses, given that poor user training may relate to poor technology acceptance [31]. Nurses received formal training in the PowerChart system away from the wards on stationary PCs, which are more comparable to laptops than tablet PCs, since navigation on tablet PCs is done using a stylus. Although tablet PCs had the longest boot-up times, this wait was not reported as a problem by clinicians and did not appear to have an influence on device selection.

### The Lack of Mobility of “Mobile” Computing Devices

Although many of the devices are intended to be mobile, it is difficult for them to match the mobility of paper [28]. Five nurses and one doctor were observed transcribing details about medication onto a napkin or paper. The “copy” of the medication chart was then used by nurses in the medication room to prepare drugs for one or several patients at a time, despite the availability of a stationary PC in the medication room. Copying details from the computerized medication chart is another example of a workaround resulting from the hardware devices failing to support adequately clinical workflow [32]. This workaround is inexpedient and can lead to errors [33]. This finding suggests that despite the necessary information being available on the stationary PC located in the medication room and users being able to log in to the PowerChart system at two different computers simultaneously, nurses find it is easier to copy information onto paper and, thus, the system is not being used as intended.

### Collaboration and Device Selection

Nurses, doctors on ward rounds, and doctors not on ward rounds collaborated mostly on the device each group preferred. Both groups of doctors collaborated when viewing results, whereas nurses mostly collaborated in order to witness the preparation of drugs. This indicates that collaboration is not the primary reason for choice of device. The study also showed that doctors mostly collaborated with other doctors, and nurses with other nurses, which is consistent with previous findings [24,34].

### Implications of the Results for Health Care Planners

The results provide some key information which may assist health planners in other hospitals to plan and evaluate their computer hardware device needs. We found that the number of COWs (five in one ward and six in the other) in each 26-bed ward appeared adequate for the tasks required. However, the number of clinical functions to be undertaken on computer devices should be considered. In our study, paper records were still used to record clinical progress notes. If this function was computerized, demand for computers would be expected to increase. Physical space limitations on a ward, including access to a power source, need to be considered when deciding upon the number of COWs which can be accommodated. This assessment of space should consider how COWs will move within patient and treatment rooms on the ward. Important features of COWs identified include the capacity to transport items such as paper charts and medications, and easy manoeuvrability. Demand for COWs was greatest during ward rounds; thus, the frequency and timing of ward rounds is a further consideration in determining the number of COWs required.

We found that despite the availability of two tablet PCs on both wards they were substantially underutilized. Interviews with nurses revealed that lack of training, specifically in the use of tablet PCs, may be a reason for nurses’ reluctance to use them more frequently. System training in hospitals usually occurs on stationary PCs with the assumption that skills will be transferable for use on other devices. Our results suggest this may not be the case, and utilization of tablet PCs might increase with specific training. Tablet PCs with handles were more frequently used, and this finding suggests that this is an important design feature to consider during hardware selection.

While there is a constant emphasis on allowing clinicians to access information at a patient’s bedside, we found that only a relatively small proportion of clinical tasks were completed at this location with more activity occurring in the corridors. This result reinforces the need for mobile devices and suggests that stationary PCs at each patient’s bedside may not be a solution to clinical information needs.

### Limitations

Our findings only relate to device selection in two wards in one hospital between the weekday hours of 7 am and 4 pm, and they may not be generalizable to other settings or times. The type of medication distribution system, namely the storage of most medicines in patients’ bedside drawers, may have influenced device preferences and, subsequently, the results. We were only able to look at devices available in the study wards and, thus, hardware devices such as personal digital assistants (PDAs) were not considered. Our hospital was still using paper-based records for some functions such as clinical progress notes. If a fully computerized patient record was in use, the need for trolley space to place paper charts would be reduced, and there would be an increased demand for access to hardware devices. Increasing the use of tablet PCs might be one solution to this demand, given that they make a reduced claim on the limited physical space available in most wards. We were only able to interview a small proportion of clinical staff to obtain data to inform the observational study results. We relied upon a convenience sample, and we cannot be sure of the representativeness of their views. However, the responses of these participants in relation to preference for specific hardware devices were consistent with the overall observational findings. We did not interview staff immediately after each observation session because this was deemed as potentially disruptive to clinical care on the ward. Additionally, only two assessors completed the heuristic evaluation, instead of the recommended minimum of three.

Data were entered into the tablet using a stylus to select from dropdown menus, press buttons, and enter text using a graphical keyboard interface on the tablet. Handwriting recognition may have been part of the tablet’s functionality, but the clinical information systems accessed using the tablet did not allow handwriting recognition. Thus, we were not able to assess this feature. The provision of specific training for nurses in the use of tablet PCs may increase their appeal.

### Conclusions

Nurses’ medication-related tasks require high levels of mobility, and computer devices need to support this mobility. Nurses move from patient to patient and back and forth between patient rooms and the medication room. All nurses preferred the generic COW independent of the clinical task conducted, particularly as it allowed them to carry other items such as paper records at the same time. However, we found evidence that mobile devices sometimes limit nurses’ mobility. In response, nurses may initiate workarounds, such as transcribing information from computer to paper rather than carrying a computer device or logging onto an available stationary PC in another location.

Doctors’ choice of device is dependent on whether or not they are on a ward round. On ward rounds, doctors move between patients and back and forth between the patient room and the corridor and, thus, require a mobile device. While mobile devices are designed to allow greater provision of information at the point of care, we found over half of observed ward round tasks were performed in corridors and away from patients’ rooms. The results indicate that a doctor’s choice of mobile device is individual and dependent on device design. Doctors not on ward rounds tend to conduct clinical tasks in the same spot, and their device of choice is the stationary PC in an office. Tablet PCs with handles were preferred to those without.

In selecting hardware devices, consideration should be given to those who will be using the system, and the nature and location of their clinical tasks, including whether ward rounds are a frequent occurrence. Furthermore, the extent to which the physical layout of a ward will accommodate different types of stationary and mobile computing devices should be considered. Devices which allow clinicians to provide care close to a patient’s bedside, but which are also easily manoeuvred to other ward locations, may reduce the initiation of potentially unsafe workaround practices.

## Abbreviations

COW: computer on wheels
CPOE: computerized provider order entry
ICT: information and communication technologies
MAR: medication administration record
PC: personal computer
PDA: personal digital assistant

## References

[1] Berg M, Winthereik BR, Berg M (2004). Waiting for Godot: episodes from the history of patient records. Health Information Management: Integrating Information Technology in Health Care Work.

[2] Anderson Gerard F, Frogner Bianca K, Johns Roger A, Reinhardt Uwe E (2006). Health care spending and use of information technology in OECD countries. Health Aff (Millwood).

[3] Häyrinen Kristiina, Saranto Kaija, Nykänen Pirkko (2008). Definition, structure, content, use and impacts of electronic health records: a review of the research literature. Int J Med Inform.

[4] Nightingale PG, Adu D, Richards NT, Peters M (2000). Implementation of rules based computerised bedside prescribing and administration: intervention study. BMJ.

[5] Richardson WC, Berwick DM, Bisgard JC, Briere R (2001). Using information technology. Crossing the Quality Chasm: A New Health System for the 21^st^ Century.

[6] Chaudhry Basit, Wang Jerome, Wu Shinyi, Maglione Margaret, Mojica Walter, Roth Elizabeth, Morton Sally C, Shekelle Paul G (2006). Systematic review: impact of health information technology on quality, efficiency, and costs of medical care. Ann Intern Med.

[7] Bardram JE (1997). I love the system — I just don’t use it. Proceedings of the International ACM SIGGROUP Conference on Supporting Group Work: The Integration Challenge (Phoenix, Arizona, USA).

[8] Han Yong Y, Carcillo Joseph A, Venkataraman Shekhar T, Clark Robert S B, Watson R Scott, Nguyen Trung C, Bayir Hülya, Orr Richard A (2005). Unexpected increased mortality after implementation of a commercially sold computerized physician order entry system. Pediatrics.

[9] Pratt Wanda, Reddy Madhu C, McDonald David W, Tarczy-Hornoch Peter, Gennari John H (2004). Incorporating ideas from computer-supported cooperative work. J Biomed Inform.

[10] Reddy MC, Dourish P, Pratt W, Printz W, Jarke M, Rogers Y, Schmidt K, Wulf V (2001). Coordinating heterogeneous work: information and representation in medical care. Proceedings of the Seventh European Conference on Computer Supported Cooperative Work.

[11] Reuss E, Menozzi M, Büchi M, Koller J, Krueger H (2004). Information access at the point of care: what can we learn for designing a mobile CPR system?. Int J Med Inform.

[12] Sellen AJ, Harper RHR (2002). The Myth of the Paperless Office.

[13] Luff P, Heath C (1998). Mobility in collaboration. Proceedings of the 1998 ACM Conference on Computer Supported Cooperative Work (Seattle, Washington, USA).

[14] Lapinsky Stephen E, Holt David, Hallett David, Abdolell Mohamed, Adhikari Neill K J (2008). Survey of information technology in Intensive Care Units in Ontario, Canada. BMC Med Inform Decis Mak.

[15] Lu Yen-Chiao, Xiao Yan, Sears Andrew, Jacko Julie A (2005). A review and a framework of handheld computer adoption in healthcare. Int J Med Inform.

[16] Ammenwerth E, Buchauer A, Bludau B, Haux R (2000). Mobile information and communication tools in the hospital. Int J Med Inform.

[17] Baumgart Daniel C (2005). Personal digital assistants in health care: experienced clinicians in the palm of your hand?. Lancet.

[18] Fischer Sandra, Stewart Thomas E, Mehta Sangeeta, Wax Randy, Lapinsky Stephen E (2003). Handheld computing in medicine. J Am Med Inform Assoc.

[19] Lindquist Anna M, Johansson Pauline E, Petersson Göran I, Saveman Britt-Inger, Nilsson Gunilla C (2008). The use of the Personal Digital Assistant (PDA) among personnel and students in health care: a review. J Med Internet Res.

[20] Lu Yen-Chiao, Xiao Yan, Sears Andrew, Jacko Julie A (2005). A review and a framework of handheld computer adoption in healthcare. Int J Med Inform.

[21] Martins HM, Jones MR (2005). What’s so different about mobile information communication technologies (MICTs) for clinical work practices? A review of selected pilot studies. Health Inform J.

[22] Westbrook Johanna I, Braithwaite Jeffrey, Georgiou Andrew, Ampt Amanda, Creswick Nerida, Coiera Enrico, Iedema Rick (2007). Multimethod evaluation of information and communication technologies in health in the context of wicked problems and sociotechnical theory. J Am Med Inform Assoc.

[23] Westbrook Johanna I, Ampt Amanda, Williamson Margaret, Nguyen Ken, Kearney Leanne (2007). Methods for measuring the impact of health information technologies on clinicians' patterns of work and communication. Stud Health Technol Inform.

[24] Westbrook Johanna I, Ampt Amanda, Kearney Leanne, Rob Marilyn I (2008). All in a day's work: an observational study to quantify how and with whom doctors on hospital wards spend their time. Med J Aust.

[25] Nielsen J (2005). Ten usability heuristics. useit.com: usable information technology.

[26] Han Sung H, Hong Sang W (2003). A systematic approach for coupling user satisfaction with product design. Ergonomics.

[27] Krogh Paul R, Rough Steve, Thomley Sylvia (2008). Comparison of two personal-computer-based mobile devices to support pharmacists' clinical documentation. Am J Health Syst Pharm.

[28] Bogossian Fiona E, Kellett Susan E M, Mason Beau (2009). The use of tablet PCs to access an electronic portfolio in the clinical setting: a pilot study using undergraduate nursing students. Nurse Educ Today.

[29] Lium Jan-Tore, Tjora Aksel, Faxvaag Arild (2008). No paper, but the same routines: a qualitative exploration of experiences in two Norwegian hospitals deprived of the paper based medical record. BMC Med Inform Decis Mak.

[30] Silvey Garry M, Lobach David F, Macri Jennifer M, Hunt Megan, Kacmaz Roje O, Lee Paul P (2006). User interface considerations for collecting data at the point of care in the tablet PC computing environment. AMIA Annu Symp Proc.

[31] Ammenwerth Elske, Gräber Stefan, Herrmann Gabriele, Bürkle Thomas, König Jochem (2003). Evaluation of health information systems-problems and challenges. Int J Med Inform.

[32] Vogelsmeier Amy A, Halbesleben Jonathon R B, Scott-Cawiezell Jill R (2008). Technology implementation and workarounds in the nursing home. J Am Med Inform Assoc.

[33] Khajouei Reza, Jaspers Monique W M (2008). CPOE system design aspects and their qualitative effect on usability. Stud Health Technol Inform.

[34] Creswick N, Westbrook JI (2008). Social network analysis of medication advice-seeking interactions among staff in an Australian hospital. Int J Med Inform.

